# Deep Conservation and Unexpected Evolutionary History of Neighboring lncRNAs MALAT1 and NEAT1

**DOI:** 10.1007/s00239-023-10151-y

**Published:** 2024-01-08

**Authors:** Forrest Weghorst, Martí Torres Marcén, Garrison Faridi, Yuh Chwen G. Lee, Karina S. Cramer

**Affiliations:** 1 Department of Neurobiology and Behavior, University of California, Irvine, USA; 2 Department of Ecology and Evolutionary Biology, University of California, Irvine, USA

**Keywords:** Long non-coding RNA, MALAT1, NEAT1, Comparative genomics, Birds

## Abstract

Long non-coding RNAs (lncRNAs) have begun to receive overdue attention for their regulatory roles in gene expression and other cellular processes. Although most lncRNAs are lowly expressed and tissue-specific, notable exceptions include MALAT1 and its genomic neighbor NEAT1, two highly and ubiquitously expressed oncogenes with roles in transcriptional regulation and RNA splicing. Previous studies have suggested that NEAT1 is found only in mammals, while MALAT1 is present in all gnathostomes (jawed vertebrates) except birds. Here we show that these assertions are incomplete, likely due to the challenges associated with properly identifying these two lncRNAs. Using phylogenetic analysis and structure-aware annotation of publicly available genomic and RNA-seq coverage data, we show that NEAT1 is a common feature of tetrapod genomes except birds and squamates. Conversely, we identify MALAT1 in representative species of all major gnathostome clades, including birds. Our in-depth examination of MALAT1, NEAT1, and their genomic context in a wide range of vertebrate species allows us to reconstruct the series of events that led to the formation of the locus containing these genes in taxa from cartilaginous fish to mammals. This evolutionary history includes the independent loss of NEAT1 in birds and squamates, since NEAT1 is found in the closest living relatives of both clades (crocodilians and tuataras, respectively). These data clarify the origins and relationships of MALAT1 and NEAT1 and highlight an opportunity to study the change and continuity in lncRNA structure and function over deep evolutionary time.

**Supplementary Information:**

The online version contains supplementary material available at 10.1007/s00239-023-10151-y.

## Introduction

The ~ 20,000 human protein-coding genes are outnumbered by the ~ 28,000 non-coding genes in the current reference assembly of the human genome (GRCh38.p14). The RNAs produced by these non-coding genes include diverse categories of small transcripts such as tRNAs, snoRNAs, piRNAs, and miRNAs, which serve a multitude of roles in gene expression and regulation (Eddy 2001; Mattick and Makunin 2006; Aalto and Pasquinelli 2012). However, the majority (~ 20,000) of human non-coding genes produce long (> 200 nt) non-coding RNAs, or lncRNAs, whose length enables greater complexity in structure and function than is possible with small RNAs (Mercer et al. 2009; Zhang et al. 2019; Statello et al. 2021; Mattick et al. 2023). LncRNAs tend to be lowly expressed and tissue specific, and many have been linked to human disease traits, reflecting their roles as regulatory molecules (Jiang et al. 2016; Kern et al. 2018; de Goede et al. 2021). A notable exception to lncRNA scarcity and specificity is the oncogene MALAT1 (Metastasis Associated Lung Adenocarcinoma Transcript), a long intergenic non-coding RNA (lincRNA) that is primarily found in nuclear speckles. Frequently among the most abundant RNAs in every tissue, MALAT1 is involved in transcriptional regulation, RNA splicing, cell division, cell death, cell differentiation, and cell migration (Zhang et al. 2012, 2017b; Gutschner et al. 2013a; Kim et al. 2018; Wang et al. 2022; Kanbar et al. 2022). While MALAT1 is expressed highly in most tissues, it is even more abundant in cancerous cells, where it is associated with chemoresistance and metastatic behavior (Gutschner et al. 2013b; Guo et al. 2015; Li et al. 2017; Xie et al. 2021; Shi et al. 2022; Hou et al. 2023). Unlike most lncRNAs, MALAT1 lacks a poly-A tail and is instead protected from exonucleases by a non-canonical 3′ arrangement: a terminal triple helix (Brown et al. 2014; Abulwerdi et al. 2019). The stretch of 9 base triplets forms after RNase P excises a downstream tRNA-like structure known as the MALAT1-associated small cytoplasmic (masc)RNA, which is co-transcribed 3′ of MALAT1 proper (Wilusz et al. 2008; Brown et al. 2012).

While the function and regulation of expression of many ancient lncRNAs are highly conserved, the primary sequence of most lncRNAs is poorly conserved across species because non-coding RNAs face no evolutionary pressure on preserving codons or reading frames (Chodroff et al. 2010; Necsulea et al. 2014; Johnsson et al. 2014; Darbellay and Necsulea 2020; Szcześniak et al. 2021; Camilleri-Robles et al. 2022). However, the secondary structure of the triple helix and mascRNA of MALAT1 has rendered these sequences resistant to mutation and has thus enabled the discovery of another unique feature of MALAT1: it is present in the genomes of all gnathostomes, from fish to humans (Stadler 2010; Zhang et al. 2017a). This sequence homology is confirmed by conserved synteny between MALAT1 and its neighboring protein-coding genes, FRMD8 and/or SCYL1. At least one of these genes borders MALAT1 in every species examined so far, and in most tetrapods, the three genes reside on the same chromosomal strand in the order FRMD8-MALAT1-SCYL1 (Stadler 2010).

Some species have a second lncRNA between FRMD8 and SCYL1, alternately denoted NEAT1 (Nuclear-Enriched Abundant Transcript) or MEN1 (Multiple Endocrine Neoplasia) (Seal et al. 2023). NEAT1 has similar characteristics and functions as MALAT1, including high abundance, enrichment in nuclear paraspeckles, rare splicing, a triple helix, a tRNA-like element known as the menRNA, and involvement in clinical outcomes of cancer (Hutchinson et al. 2007; Brown et al. 2012; Hu et al. 2018; Shin et al. 2019; Pisani and Baron 2020; Knutsen et al. 2022). One difference between MALAT1 and NEAT1 is the presence of an internal poly-A signal in NEAT1, which enables the transcription of a short (~ 3 kb in humans) poly-adenylated isoform called MENε in addition to the long (~ 20 kb in humans) triple-helicate isoform known as MENβ (Stadler 2010; Naganuma et al. 2012; Isobe et al. 2020; Knutsen et al. 2022). By contrast, MALAT1 primarily exists as one triple-helicate isoform (~ 7 kb in humans). These similarities and the genomic proximity of MALAT1 and NEAT1 suggest the possibility that they arose due to a duplication of all or part of the ancestral MALAT1 gene. NEAT1 has historically been considered a mammalian innovation (Stadler 2010), but recent studies have cast doubt on this idea by identifying multiple triplex-masc/menRNA motifs in the genomes of non-mammalian tetrapods (Zhang et al. 2017a), highlighting a more general problem of poor lncRNA annotation in most genome assemblies (Necsulea et al. 2014; Darbellay and Necsulea 2020). The antiquity and ubiquity of MALAT1 and NEAT1 thus provide a rare opportunity to study lncRNA evolution across a wide range of taxa.

Surprisingly, neither NEAT1 nor MALAT1 has been described in any avian species. Some have surmised that birds either lost the locus or, more likely, that the genes reside on one of birds’ ~ 30 microchromosomes (Stadler 2010), which have poor coverage in genome assemblies, likely due to their small size and high GC content (Srikulnath et al. 2021; Waters et al. 2021; Li et al. 2022). More recent studies identified a MALAT1/NEAT1-like triple helix in the genomes of several bird species (Sun et al. 2017; Zhang et al. 2017a), but the fragmentary nature of avian genome assemblies at the time precluded further characterization of the associated genes, which spanned multiple genomic scaffolds. Here we provide evidence that MALAT1, but not NEAT1, is present in the genomes of several dozen bird species, where it has been revealed thanks to recent advances in sequencing technology (Rhie et al. 2021). We verify that avian MALAT1 resembles MALAT1 orthologs of other vertebrates in its conservation of the triple helix, conservation of gene order with FRMD8 and SCYL1, and high expression level. We use phylogenetic analysis and RNA-seq coverage data to demonstrate that the avian gene likely descended from MALAT1 (not NEAT1). We also show that other reptiles have NEAT1, suggesting that avian ancestors lost NEAT1 after they diverged from crocodilian ancestors. Finally, we present a model of major events in the evolutionary history of MALAT1 and NEAT1, including the establishment of conserved synteny of ancestral MALAT1 with FRMD8 and SCYL1 as well as the likely (whole or partial) duplication of this ancestral gene into genes that became the MALAT1 and NEAT1 seen in tetrapods.

## Results

### Identification of Avian MALAT1

**Fig. 1:**
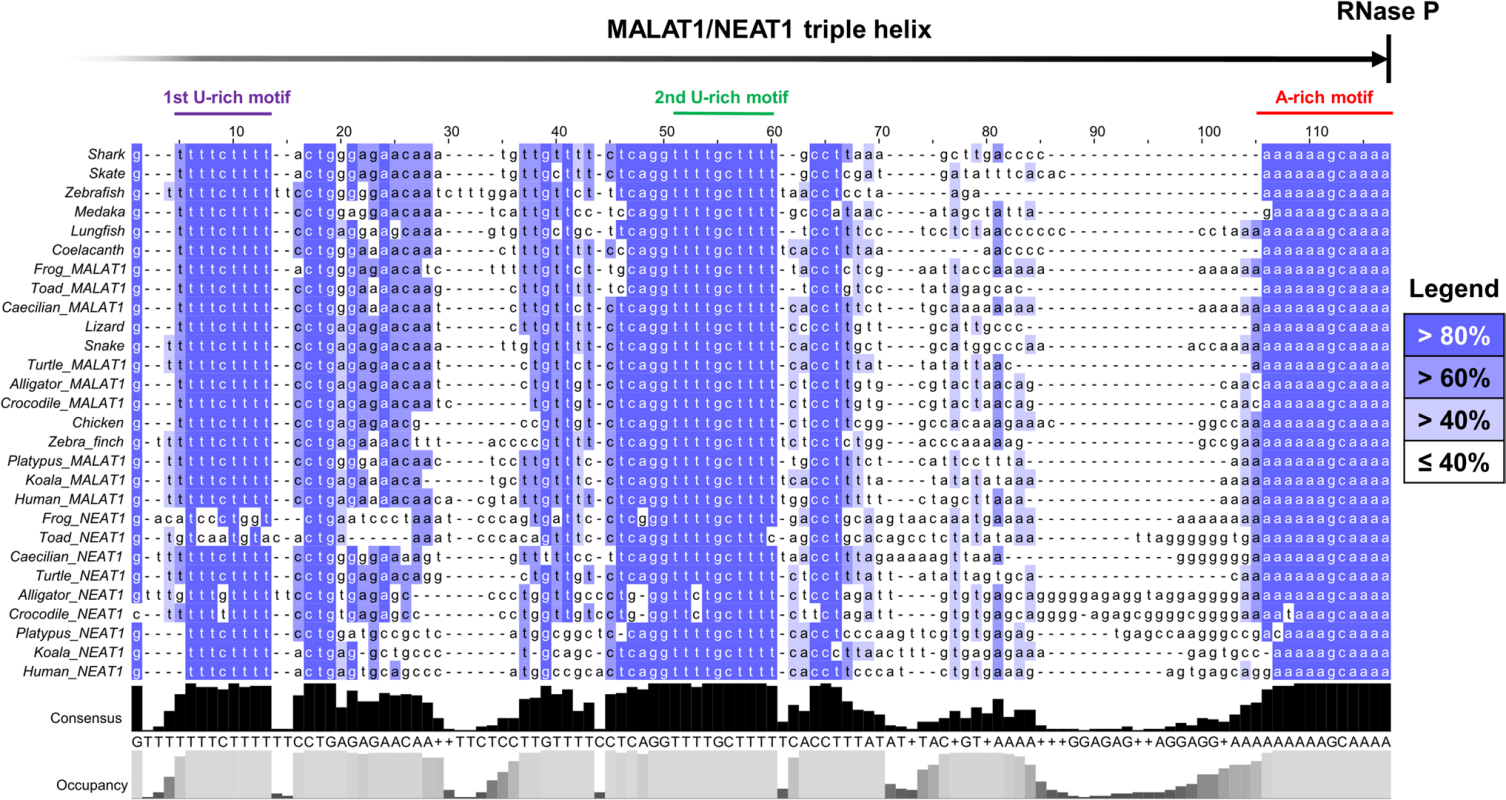
The nucleotide sequence of the avian triple helix resembles that of other vertebrate MALAT1 and NEAT1 orthologs. BLAST hits of the triple helix in chicken and zebra finch were aligned to 3′ termini of MALAT1 and NEAT1 genes previously described in other vertebrates. Strong primary sequence conservation is observed, especially in the regions that participate in secondary structure, namely the U-rich and A-rich components of the triple helix. The RNase P cleavage site, which defines the ends of the MALAT1 and NEAT1 transcripts, is indicated by a vertical line on the gene legend. Nucleotides are colored in different tiers according to their percent identity with other bases in the same position. Percent identity thresholds are indicated by the legend. Figure created with Jalview v2.11.2.7

We identified candidates for avian orthologs of MALAT1/NEAT1 in two stages. First we used NCBI BLAST (Madden 2003; Johnson et al. 2008) to search the chicken genome for the human MALAT1 triple helix and mascRNA sequence (“TripHelMasc”), which yielded one highly significant hit (E-value = 7 × 10^–11^). Then we used the sequence of this hit to query all avian genomes. The search returned hits in 37 avian species (E < 1 × 10^–7^), all of which resembled known triple helix sequences in non-avian species (Fig. 1; Supplementary Data 1). For each avian species, we used the NCBI Genome Data Viewer (Sayers et al. 2023) to manually annotate the gene corresponding to each BLAST hit (Supplementary Data 1). MALAT1/NEAT1 orthologs are clearly discernible with the “RNA-seq exon coverage” track, as these genes’ expression levels far exceed those of nearby transcribed regions (Supplementary Fig. 1). Moreover, the avian orthologs begin 25–35 bp downstream of a canonical TATA box, and they terminate at the triple helix, so these features were used to define the gene start and end coordinates, respectively, in the genomic areas where RNA-seq coverage drops off.

RNA-seq coverage data also provided evidence that the avian orthologs were more closely related to MALAT1 than to NEAT1: RNA-seq coverage level was approximately constant over the extent of the gene, consistent with RNA-seq coverage data for MALAT1 in other tetrapods and reflecting a single isoform of the gene (Supplementary Fig. 1H–S). By contrast, NEAT1 orthologs in tetrapods have a very high RNA-seq coverage level at the 5′ end of the gene in the region shared by the two NEAT1 isoforms, followed by a lower coverage level in the 3′ portion of the gene, which is only found in the longer MENβ transcript (Supplementary Fig. 1H–J, M–N, Q–S). It remains possible that the avian gene descended from a NEAT1 ortholog that no longer generates the shorter MENε transcript, but here we refer to the avian gene as MALAT1 for simplicity. Complete MALAT1 orthologs were identified in 14 of the 37 bird genomes with TripHelMasc BLAST hits (Supplementary Data 1). Of the remaining 23 genomes, 13 MALAT1 orthologs were incomplete because they contained stretches of unknown nucleotides (“Ns”), and 10 were incomplete because the scaffold sequence ended before the MALAT1 sequence did. However, because of the strong conservation of the distinctive TripHelMasc sequence and of very high RNA-seq expression level even in the partially assembled orthologs, all 37 of these loci are strong candidates for MALAT1 orthologs and will likely be completed as sequencing efforts expand. These results suggest the presence of a single MALAT1-like gene in each avian genome.

### Avian MALAT1 Is Neighbored by the Same Protein-Coding Genes as in Other Vertebrates

We next sought to determine whether FRMD8 and SCYL1 neighbor the avian candidate MALAT1 orthologs, as they do in other vertebrates. We used NCBI Genome search (Sayers et al. 2023) to query FRMD8 and SCYL1 in birds, and we compared the scaffold, strand, and position of each neighboring gene to the scaffold, strand, and position of each candidate MALAT1 ortholog (Supplementary Data 1). Of the 24 bird genomes with FRMD8, 21 also have a TripHelMasc BLAST hit, and 20 of these are on the same scaffold and strand as the MALAT1 gene in each species, with the FRMD8 start position just a few kb 5′ of the MALAT1 start position (Supplementary Data 1). Similarly, of the 17 bird genomes with SCYL1, 15 also have a TripHelMasc BLAST hit, all of which are on the same scaffold and strand as MALAT1, with SCYL1 start positions a few kb 3′ of MALAT1 (Supplementary Data 1). These loci match the gene order of FRMD8, MALAT1, and SCYL1 in other species, further suggesting that avian MALAT1 candidates are true orthologs.

Five avian species had either FRMD8 or SCYL1 without a BLAST hit for the TripHelMasc sequence. In three of these species, the scaffold with the protein-coding gene ended before the MALAT1 sequence was expected to begin, suggesting MALAT1 lies in an unassembled region in these genomes. However, in the other two species (white wagtail and New Caledonian crow), MALAT1 was absent despite sufficient genomic space on the scaffold. Notably, either FRMD8 or SCYL1 was also absent in each of these species, so it is likely that a chromosomal translocation broke the synteny at the MALAT1 locus. Both MALAT1 and the missing protein-coding gene will likely be uncovered by future efforts to improve genome assembly.

### Avian MALAT1 Is More Closely Related to MALAT1 Than NEAT1 in Other Tetrapods

While some have suggested that NEAT1 is a mammal-specific gene (Stadler 2010), recent studies have challenged this view by showing that other tetrapods consistently have at least two genomic hits for the TripHelMasc/Men sequence, potentially corresponding to MALAT1 and NEAT1 (Zhang et al. 2017a). However, we observed a single unique hit per species for the TripHelMasc/Men sequence in all avian genomes, even with more extensive BLAST searches of the avian nucleotide collection (nr/nt) and whole genome sequencing contig (wgs) databases, suggesting that birds only have one of the two triple-helicate lncRNAs.

We therefore sought to determine whether this single-gene arrangement is an ancestral or derived trait in birds, and to identify which of the two genes is the likely ancestor of the avian orthologs. We characterized MALAT1 and NEAT1 genes in several tetrapod species representative of major clades (Supplementary Data 2; Supplementary Fig. 1). Notably, MALAT1 and NEAT1 were only correctly annotated in manually annotated genomes (i.e., only human among the species we considered), suggesting a shortcoming in the ability of automated genome annotation pipelines to identify these lncRNAs. We therefore used the same strategy as for birds to find MALAT1 and NEAT1: a BLAST search for the human TripHelMasc sequence in the RefSeq Representative Genomes for each taxon, followed by manual inspection of RNA-seq coverage data surrounding BLAST hits in the Genome Data Viewer. MALAT1 and NEAT1 were differentially identified using the RNA-seq coverage patterns described above: High 5′ coverage and lower 3′ coverage for NEAT1, and consistent coverage for MALAT1.

**Fig. 2:**
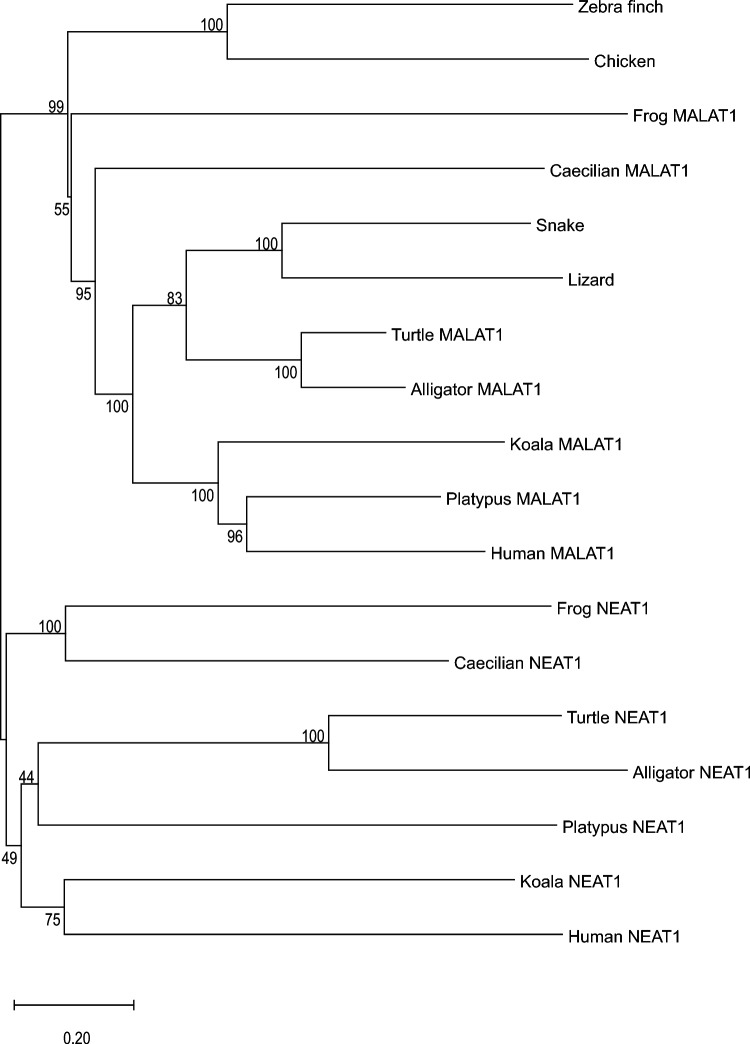
Phylogenetic analysis of MALAT1 and NEAT1 sequences reveals that the avian ortholog is descended from MALAT1, not NEAT1. Full MALAT1 and NEAT1 gene sequences of species representing major tetrapod taxa were aligned with MAFFT, and a maximum-likelihood phylogenetic tree was constructed with MEGA11. MALAT1 and NEAT1 orthologs were segregated with 99% bootstrap support, corroborating our method of distinguishing the two genes based on RNA-seq coverage data. Each gene’s clade generally matches the true relationships of the species examined, except the avian MALAT1 orthologs are unexpectedly rooted prior to the divergence of tetrapod MALAT1. Numbers on nodes indicate the percentage of bootstrap support for each clade, based on 100 replicates. Scale bar denotes genetic distance per branch length in number of substitutions per site

We found MALAT1 and NEAT1 in all major tetrapod taxa except birds and squamates (lizards and snakes), both of which had a single gene with an RNA-seq expression profile that resembled that of MALAT1 (Supplementary Fig. 1K–S; Supplementary Data 2). To further interrogate the ancestry of avian MALAT1, we aligned full MALAT1 and NEAT1 gene sequences from these representative tetrapod species (including chicken and zebra finch) and constructed a phylogenetic tree (Fig. 2) via the maximum likelihood method of MEGA11 (Tamura et al. 2021). The resulting tree generally matched the true phylogeny of tetrapods for both genes, and the node separating MALAT1 from NEAT1 orthologs received 99% bootstrap support. This result corroborates the identity of the avian and squamate orthologs as MALAT1, since both clades were placed in the MALAT1 half of the phylogenetic tree. Puzzlingly, the avian clade was rooted prior to the divergence of tetrapod MALAT1 rather than in its expected position in the reptilian MALAT1 clade, with alligator MALAT1 as its closest relative. This aberrant placement suggests that avian MALAT1 underwent rapid evolution after the divergence of avian and crocodilian ancestors. Nevertheless, these results suggest that birds and squamates lost NEAT1 and kept MALAT1.

### Evolutionary History of MALAT1 and NEAT1

**Fig. 3:**
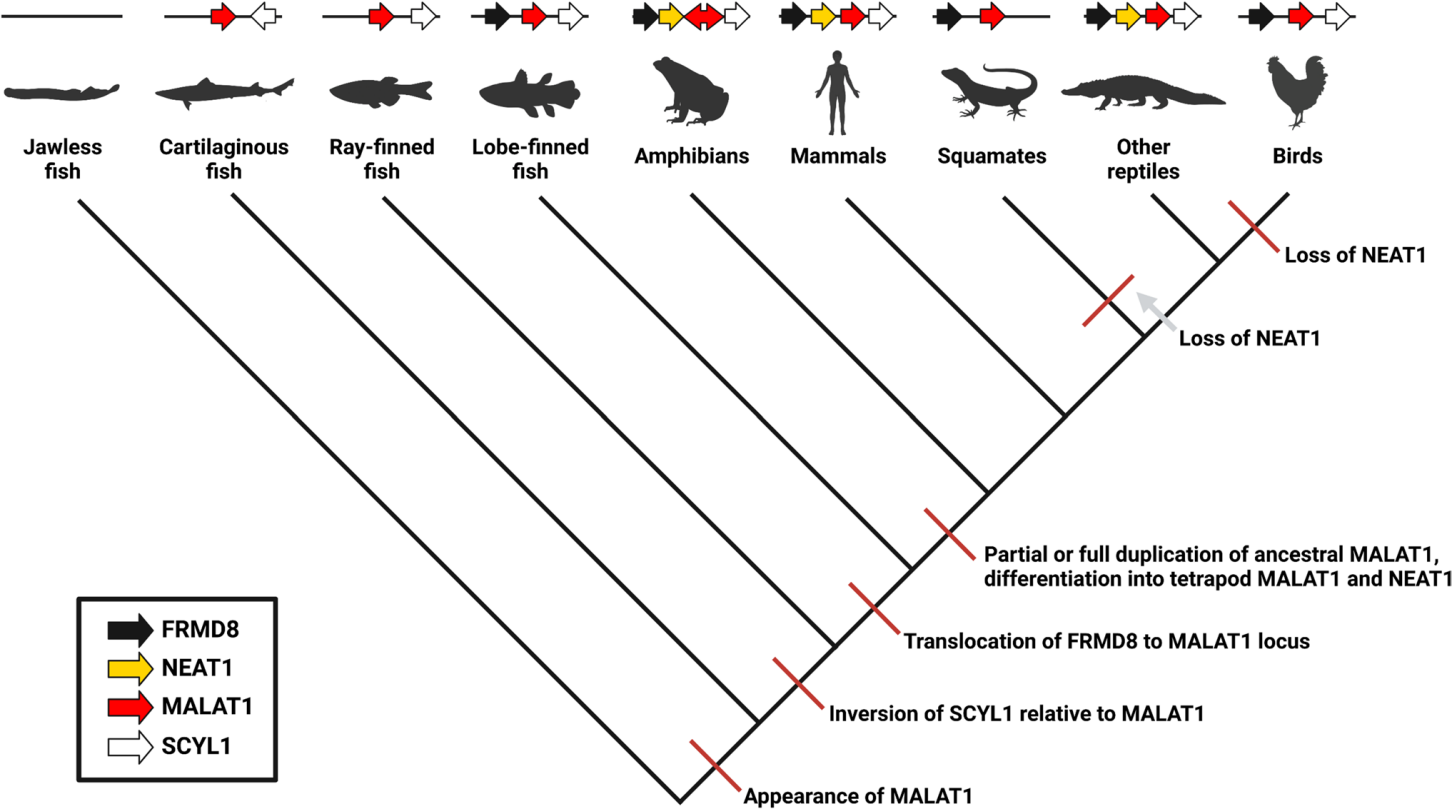
Cladogram of major events in the evolutionary history of MALAT1 and NEAT1. To reconstruct the evolutionary history of MALAT1 and NEAT1, orthologs were identified in representative vertebrate clades by BLAST search of the TripHelMasc sequence, followed by characterization of the genomic region surrounding these genes. MALAT1 first appeared prior to the divergence of cartilaginous and bony fish, and a chromosomal inversion and translocation resulted in the tetrapod-like configuration of FRMD8-MALAT1-SCYL1 prior to the divergence of coelacanths and tetrapods. At least part of the ancestral MALAT1 was duplicated prior to the radiation of tetrapods, yielding MALAT1 and NEAT1. Finally, NEAT1 was lost in squamates and birds independently. Gene diagrams above the species silhouettes indicate both order and direction (5′–3′) of the genes, which are color-coded according to the legend. Amphibian MALAT1 is depicted by a double-headed arrow because MALAT1 is on a different strand from the other three genes in frogs and toads, but this inversion appears to be lineage-specific, as all four genes are on the same strand in other amphibians (i.e., caecilians). Figure created with Biorender.com

Our findings enable amendment of the current understanding of MALAT1 and NEAT1 ancestry. To supplement the results of the phylogenetic analysis and further illuminate the major events in the evolutionary history of these genes, we characterized MALAT1 in species of fish representative of major taxa using the same methods mentioned above (Supplementary Fig. 1, Supplementary Data 2). We found that jawless fish (lampreys) do not have MALAT1, but all jawed fish have a single MALAT1-like gene (which we will call MALAT1 for simplicity, since its RNA-seq coverage profile matches that of tetrapod MALAT1), suggesting that MALAT1 originated after the divergence of jawed and jawless vertebrates at least 500 million years ago (Ma) but before the divergence of *Chondrichthyes* (cartilaginous fish) and *Osteichthyes* (bony fish) around 439 Ma (Fig. 3) (Zhu et al. 2009; Brazeau and Friedman 2015; Yang et al. 2018). Additionally, the likely series of events in the assemblage of the tetrapod MALAT1 locus can be inferred from the conserved synteny in major fish clades (Fig. 3). In *Chondrichthyes*, MALAT1 and SCYL1 are on different DNA strands with adjacent 3′ ends, while FRMD8 is not present at the locus. In *Actinopterygii* (ray-finned fish), MALAT1 and SCYL1 are on the same strand, and the 3′ end of MALAT1 is adjacent to the 5′ end of SCYL1, as in tetrapods. FRMD8 is not present at the locus in this clade either. However, in some members of *Sarcopterygii* (lobe-finned fish), the tetrapod gene order (FRMD8-MALAT1-SCYL1) is apparent, with all genes on the same strand. Thus, oppositely-stranded MALAT1 and SCYL1 is the most ancestral condition discernible with extant genomes. A chromosomal inversion placed MALAT1 and SCYL1 on the same strand after the divergence of *Chondrichthyes* and *Osteichthyes* but before the divergence of *Actinopterygii* and *Sarcopterygii* (i.e., between 439 and 425 Ma) (Zhu et al. 2009; Brazeau and Friedman 2015). Finally, FRMD8 was translocated to the 5′ end of the MALAT1-SCYL1 locus after the divergence of *Actinopterygii* and *Sarcopterygii* but before the divergence of *Actinista* (coelacanth ancestors) and *Tetrapoda* (i.e., between 425 and 409 Ma; Fig. 3) (Lu et al. 2012; Zhao et al. 2021).

As previously discussed, we found that all tetrapods examined have MALAT1, and all have NEAT1 as well except birds and squamates (lizards and snakes). NEAT1 is thus a tetrapod-specific (not a mammal-specific) gene, and the tetrapodal MALAT1 and NEAT1 genes likely originated from a duplication of all or part of the ancestral gene after the rise of tetrapods but before the divergence of amphibians and amniotes (i.e., between 409 and 330 Ma; Fig. 3) (Benton and Donoghue 2007; Lu et al. 2012; Zhao et al. 2021). Notably, the closest living relatives of birds and squamates (crocodilians and tuataras (Gemmell et al. 2020), respectively) have NEAT1, suggesting that the ancestor of modern birds lost NEAT1 between 237 and 70 Ma (Prum et al. 2015; Claramunt and Cracraft 2015), and that this happened independently of the loss of NEAT1 in the ancestor of squamates, which occurred between 250 and 232 Ma (Fig. 3) (Evans and Jones 2010; Jones et al. 2013; Burbrink et al. 2020; Whiteside et al. 2022).

## Discussion

Here we have shown for the first time that MALAT1, a highly expressed lncRNA with diverse regulatory functions in a variety of cell types, is conserved in birds. We build upon previous publications that predicted the presence of avian MALAT1 but were unable to present a full gene sequence or investigate other features of the transcript in question because of the incompleteness of avian genomes at the time of publication (Stadler 2010; Sun et al. 2017; Zhang et al. 2017a). Full characterization of the MALAT1 gene, as we have done here, is essential because detection of a MALAT1-like triple helix and mascRNA does not prove the existence of a MALAT1 ortholog. Indeed, one of these studies described MALAT1-like TripHelMasc elements in dozens of transcripts in anole lizards and in some species of fish, suggesting that the MALAT1 triple helix was duplicated and inserted elsewhere in the genome (Zhang et al. 2017a). Even though birds only have a single copy of the TripHelMasc sequence, the presence of NEAT1 in some reptiles called into question whether the corresponding avian gene is MALAT1 or NEAT1. We therefore characterized the phylogeny of MALAT1 and NEAT1 in tetrapods and found that the single triple helicate gene in birds and squamates is likely descended from an ancestral MALAT1, not NEAT1. Finally, we characterized the evolutionary history of the MALAT1/NEAT1 locus and identified likely timeframes for the major events in the construction of this locus.

### Origin of NEAT1

An atypical situation is observed in *Anura* (frogs and toads): The lncRNA closer to SCYL1 is on the opposite strand from the other three genes at the locus (Supplementary Fig. 1H and I). This arrangement led to a previous interpretation that "the frog genome contains two divergent, and hence ancient, copies of MALAT1 in an unexpected tail-to-tail configuration. The phylogenetic analysis does not provide any evidence that one of these copies might be the ancestor of [NEAT1]” (Stadler 2010). Here we present evidence that contradicts this assertion, showing that amphibians indeed have MALAT1 and NEAT1. The example of caecilians is instructive, as these legless amphibians have a MALAT1 locus identical to that found in most amniotes (Supplementary Fig. 1J). Two abundant triple-helicate lncRNAs reside between and on the same strand as FRMD8 and SCYL1. The lncRNA closer to FRMD8 has an RNA-seq coverage profile resembling that of NEAT1 (higher 5′ expression, lower 3′ expression), while the lncRNA closer to SCYL1 resembles MALAT1 (consistent expression across the transcript). The most parsimonious explanation of this observation is that NEAT1 appeared in the tetrapod lineage prior to the common ancestor of amphibians and amniotes, and the partially inverted locus found in anurans is a derived trait. Our phylogenetic analysis (Fig. 2) confirms the assignment of amphibian MALAT1 and NEAT1 by RNA-seq coverage data, as the amphibian genes segregate with their amniote relatives, with 99% bootstrap support for the MALAT1-NEAT1 schism in tetrapods. We suspect our phylogenetic analysis yielded different results than Stadler’s because instead of ClustalW, we used the E-INS-I algorithm of MAFFT to align MALAT1 and NEAT1 sequences. This algorithm is better suited to large alignments with multiple unaligned regions (Katoh et al. 2002; Katoh and Toh 2008), as occurs with MALAT1 and NEAT1 in distantly related species. We also included a greater diversity of tetrapod MALAT1 and NEAT1 genes with which the amphibian genes might find better alignment.

The fact remains that primary sequence comparison is a fraught method for determining lncRNA relatedness. Future studies may use more detailed information, such as microsynteny and secondary structure, to make stronger claims about homology of MALAT1 and NEAT1 in distantly related species, as has already been attempted in several cases of more closely related species for these lncRNAs (Andrews et al. 2017; Lin et al. 2018; McCown et al. 2019; Walter Costa et al. 2019; Monroy-Eklund et al. 2023) and other RNAs (Tavares et al. 2019; Herrera-Úbeda et al. 2019; Morandi et al. 2022). However, this expectation should be tempered, as critical re-examinations of some of these studies on ancient lncRNAs have found little to no evidence of homology even at the level of secondary structure, in part because previous claims of homology were obtained by the misuse of bioinformatic and statistical methods typically employed to infer structural motif conservation (Rivas et al. 2017; Rivas and Eddy 2020; Rivas 2021, 2023; Gao et al. 2023).

### Divergence of MALAT1 Sequence in Taxa That Lost NEAT1

We were surprised to find that avian MALAT1 has changed so thoroughly from its ancestral form that phylogenetic analysis of full gene sequences failed to group it with crocodilian MALAT1 (Fig. 2). One possible explanation for this observation is that loss of NEAT1 is associated with distinct selective pressure on MALAT1, resulting in compensatory evolution of MALAT1, as has been shown in the loss of paralogs of protein-coding genes (Albalat and Cañestro 2016). Perhaps MALAT1 underwent mutations that rendered some functions of NEAT1 redundant. This may have relaxed the selective constraints on NEAT1 and eventually led to the loss of the gene. The sequence (and thus the function) of the resulting MALAT1 could differ greatly both from ancestral and extant MALAT1 genes, which would explain why avian MALAT1 is rooted at the base of the tetrapod MALAT1 branch (Fig. 2). The process of compensatory evolution after gene deletion, especially in paralogs of deleted genes, has been extensively studied in yeast (Szamecz et al. 2014; Echenique et al. 2019; Helsen et al. 2020; Farkas et al. 2022). In addition, several examples in vertebrates (Cañestro et al. 2009, 2013; Thompson et al. 2016) suggest plausible mechanisms for changes in MALAT1 in taxa that have lost NEAT1. Since MALAT1 and NEAT1 have functional and structural similarities, the principles of genetic change in response to paralog loss could apply to NEAT1 loss even if tetrapod MALAT1 is not a full paralog of tetrapod NEAT1. A possible objection to this argument is that squamate MALAT1 was correctly placed within the phylogenetic tree, despite squamates also losing NEAT1. The differences between MALAT1 in these two clades will likely be a fruitful resource for future studies on lncRNA evolution.

There is some evidence that the process of NEAT1 loss in avian ancestors had begun prior to the divergence of birds and crocodilians. While crocodilian NEAT1 is discernible based on its RNA-seq coverage pattern as described above, it is abnormal in two regards: it is much shorter (~ 7 kb; Supplementary Fig. 1N) than the average NEAT1 gene (> 20 kb), and its 3′ terminus has several mutations in nucleotides that are typically involved in the NEAT1 triple helix and that are invariant in most other species examined (Fig. 1), both of which suggest decreased pressure of purifying selection on NEAT1. These differences are present in both alligators and crocodiles (Ghosh et al. 2020), whose most recent common ancestor lived 80 to 90 Ma (Brochu 2003). Though this evidence shows that changes to NEAT1 had occurred prior to the radiation of crocodilians, other findings suggest that these changes happened earlier. The crocodilian rate of molecular evolution has been estimated as the lowest of all living amniotes, and since archosaurs’ sister clade (turtles) mutate almost as slowly as crocodilians, slow molecular evolution is thought to represent the ancestral condition of archosaurs (Green et al. 2014). The differences in crocodilian NEAT1 thus may reflect the initial events in the archosaur lineage that culminated in the loss of NEAT1 and the relatively rapid compensatory evolution of MALAT1 in birds, whose lineage-specific rate of mutation is four times that of crocodilians (Green et al. 2014).

### Recommendations for MALAT1 and NEAT1 Annotation

Several unorthodox features of MALAT1 and NEAT1 have hindered their identification by automated pipelines used to annotate genes in the most commonly used genome assemblies, including NCBI RefSeq, Ensembl, and UCSC. We were surprised to find that only species with manually curated genomes (such as human and mouse) have correct annotations for MALAT1 or NEAT1 in any of these databases, despite the known ubiquity of these genes throughout the literature on them (Stadler 2010; Zhang et al. 2017a; McCown et al. 2019). LncRNAs are inherently difficult to annotate, as their poor sequence conservation and lack of open reading frames inhibits gene assignment by homology. MALAT1 and NEAT1 are particularly prone to misannotation because they lack introns, which are used to distinguish transcriptional noise from functional transcription, and because they have terminal triple-helices instead of poly-A signal sequences, which can aid in defining the 3′ ends of genes. MALAT1 and NEAT1 are then either not annotated at all, or they are terminated prematurely at a random internal poly-A signal sequence (Supplementary Fig. 1). Downstream elements such as the TripHelMasc/Men sequence are thus overlooked, even though their homology with annotated transcripts may enable gene identification. We recommend an annotation step for vertebrate genomes to ensure that future assemblies include MALAT1 and NEAT1. Because these genes are so atypical, they may require a dedicated step in gene annotation pipelines. A strong candidate resembles our strategy here, namely defining the 3′ end of the gene at a BLAST hit for the TripHelMasc sequence and the 5′ end with transcription start site expression data if available, or with a combination of decreased RNA-seq expression level and proximity to a TATA box. As knowledge of lncRNA structure and homology expands, similar methods may be necessary to improve the inclusion of other conserved lncRNAs in genome annotations (Salzberg 2019; Rhie et al. 2021).

### Future Directions and Conclusion

MALAT1 and NEAT1 are two of the most highly conserved and abundantly expressed lncRNAs in vertebrates. Their overexpression in several types of human cancer and their implication in chemoresistance (Li et al. 2017; Hu et al. 2018; Shin et al. 2019; Pisani and Baron 2020; Hou et al. 2023) emphasizes the need to better understand the normal and pathological functions of these lncRNAs. Since conservation of lncRNAs is rare, MALAT1 and NEAT1 provide a unique opportunity for studies on comparative genetics and genomics to address a variety of questions about their function, and more broadly about the consequences of gene duplication and loss for non-coding genes: What selective pressure (positive or negative) governs the evolution of MALAT1 and NEAT across species? How did the appearance of NEAT1 in tetrapods and its subsequent loss in birds and squamates alter the sequence and functional evolution of MALAT1 in these respective taxa? How do the evolutionary changes of MALAT1 and NEAT1 influence the evolution of the proteins and RNA that interact with them? We anticipate that the research addressing these questions will illuminate the functions of MALAT1 and NEAT1 more thoroughly than studies on these genes in a single species, highlighting the importance of an evolutionary and comparative foundation even for fields with a distinct clinical application.

## Methods

### Identification and Characterization of MALAT1 and NEAT1

The sequence of the human MALAT1 triple helix and mascRNA were used as a query for a blastn search of the chicken reference genome (GRCg7b) on NCBI BLAST (Madden 2003; Johnson et al. 2008) with otherwise default settings. The most significant hit (which was considered the likely sequence of the chicken MALAT1 triple helix and mascRNA) was used as a blastn query of Aves Refseq genomes to locate MALAT1 in other birds. The NCBI Genome Data Viewer (Sayers et al. 2023) was then used to evaluate coordinates, expression levels, splice sites, and conserved synteny of the identified MALAT1 ortholog candidates. The same process was used to identify MALAT1 and NEAT1 orthologs in representative vertebrate taxa. The two genes were distinguished using their RNA-seq coverage patterns: high 5′ coverage and low 3′ coverage for NEAT1, and consistent coverage for MALAT1. This criterion was validated by the phylogenetic analysis in Fig. 2, which separated all MALAT1 orthologs from all NEAT1 orthologs with 99% bootstrap support. For tetrapod taxa in which NEAT1 was not identified on the first pass (i.e., birds and squamates), blastn searches of whole genome sequencing contigs (wgs) and the nucleotide collection (nr/nt) were conducted with the nearest living relative’s TripHelMen sequence as bait to rule out the possibility that NEAT1 had translocated elsewhere in the genome. While tuatara (Gemmell et al. 2020) does not currently have sufficient RNA-seq coverage data to identify NEAT1 and MALAT1 in the way we did with other tetrapods, the Green Anole triple helix returned exactly two BLAST hits in the tuatara genome, both within a few kilobases of each other on the same scaffold. This finding was taken as evidence that tuataras have both lncRNAs, and squamates lost NEAT1 after they diverged from the ancestors of tuataras.

### Phylogenetic Analysis

Evolutionary conservation of the MALAT1 gene was explored by using the E-INS-I algorithm of MAFFT v7.505 to align full MALAT1 and NEAT1 gene sequences of tetrapods listed in Supplementary Data 2 (except the Common toad, *Bufo bufo*, because its NEAT1 gene is too long: 116 kb). This alignment was used for phylogenetic tree reconstruction via the maximum likelihood method in MEGA11 (Tamura et al. 2021). The Bayesian Information Criterion was used to aid substitution model selection for the phylogenetic analysis: Generalized Time Reversible model with 5 gamma categories, invariant sites, 100 bootstrap replicates, and all sites considered. Triple helices of the same species plus common toad, tuatara (Gemmell et al. 2020), and saltwater crocodile (Ghosh et al. 2020) were aligned with MAFFT E-INS-I and manually curated in Jalview v2.11.2.7 to create Fig. 1.

### Supplementary Information

Below is the link to the electronic supplementary material.

Supplementary Data 1 Information on MALAT1 in avian species
Supplementary file1 (XLSX 19 KB)

Supplementary Data 2 Information on MALAT1 and NEAT1 in species representative of major vertebrate clades
Supplementary file2 (XLSX 20 KB)

Supplementary Data 3 SRA Accessions of RNA-seq data that contributed to coverage data in Supplementary Fig. 1
Supplementary file3 (ZIP 608 KB)

Supplementary Fig. 1. RNA-seq coverage data of MALAT1 and NEAT1 in species representative of major vertebrate taxa. The genomic neighborhood surrounding MALAT1 and NEAT1 in species representative of major vertebrate taxa was exported from NCBI Genome Data Viewer. Tracks included current gene definitions in NCBI RefSeq and Ensembl genome assemblies, as well as RNA-seq alignment coverage. Arrows beneath the RNA-seq coverage track indicate the locations and strand of MALAT1 (red arrow), NEAT1 (orange arrow), and FRMD8 and/or SCYL1 (black arrows). For MALAT1 and NEAT1, the location of the arrowhead indicates the approximate location of the triple helix. The distinction between MALAT1 and NEAT1 can be observed in their RNA-seq coverage patterns for the species that have both. RNA-seq experiments that contributed to the coverage data for each species can be found in Supplementary Data 3
Supplementary file4 (PDF 1648 KB)
