# Supplementary material for: Deep Conservation and Unexpected Evolutionary History of Neighboring lncRNAs MALAT1 and NEAT1
Source: J Mol Evol. 2024 Jan 8;92(1):30–41. doi: 10.1007/s00239-023-10151-y (PMC10869381; doi:10.1007/s00239-023-10151-y)

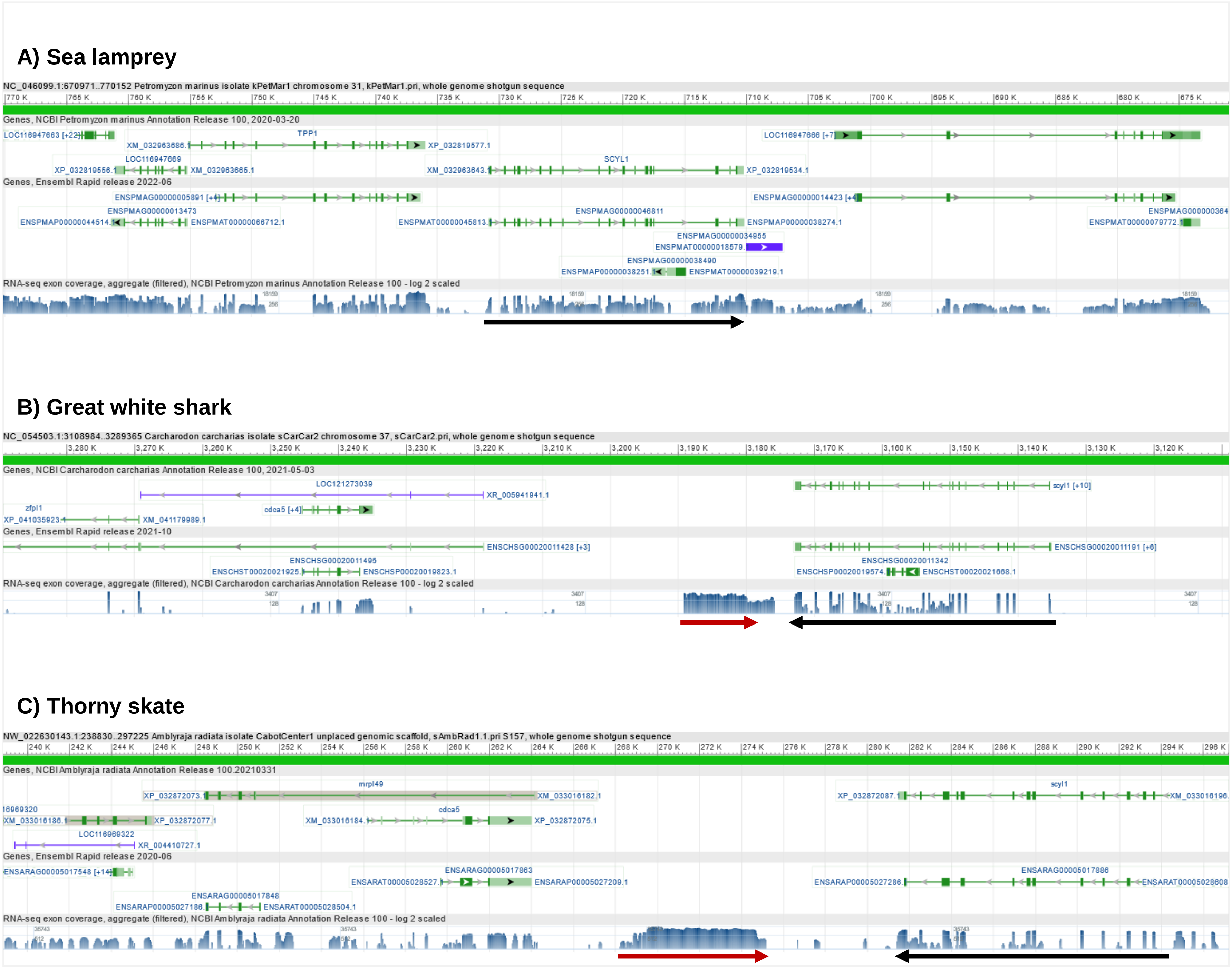

A) Sea lamprey
NC_046099.1:670971..770152 Petromyzon marinus isolate kPetMar1 chromosome 31, kPetMar1.pri, whole genome shotgun sequence
Genes, NCBI Petromyzon marinus Annotation Release 100, 2020-03-20
LOC116947663 [+22]
TPP1
XM_032963686.1
XP_032819577.1
LOC116947666 [+7]
LOC116947669
XP_032819556.1
XM_032963665.1
SCYL1
XM_032963643.1
XP_032819534.1
Genes, Ensembl Rapid release 2022-06
ENSPMAG00000005891 [+4]
ENSPMAG00000014423 [+4]
ENSPMAG00000013473
ENSPMAP00000044514.1
ENSPMAT00000066712.1
ENSPMAG00000046811
ENSPMAT00000045813.1
ENSPMAP00000038274.1
ENSPMAT00000079772.1
ENSPMAG00000034955
ENSPMAT00000018579.1
ENSPMAG00000038490
ENSPMAP00000038251.1
ENSPMAT00000039219.1
RNA-seq exon coverage, aggregate (filtered), NCBI Petromyzon marinus Annotation Release 100 - log 2 scaled
B) Great white shark
NC_054503.1:3108984..3289365 Carcharodon carcharias isolate sCarCar2 chromosome 37, sCarCar2.pri, whole genome shotgun sequence
Genes, NCBI Carcharodon carcharias Annotation Release 100, 2021-05-03
LOC121273039
XR_005941941.1
scyl1 [+10]
zfpl1
XP_041035923.1
XM_041179989.1
cdca5 [+4]
Genes, Ensembl Rapid release 2021-10
ENSCHSG00020011428 [+3]
ENSCHSG00020011191 [+6]
ENSCHSG00020011495
ENSCHST00020021925.1
ENSCHSP00020019823.1
ENSCHSG00020011342
ENSCHSP00020019574.1
ENSCHST00020021668.1
RNA-seq exon coverage, aggregate (filtered), NCBI Carcharodon carcharias Annotation Release 100 - log 2 scaled
C) Thorny skate
NW_022630143.1:238830..297225 Amblyraja radiata isolate CabotCenter1 unplaced genomic scaffold, sAmbRad1.1.pri S157, whole genome shotgun sequence
Genes, NCBI Amblyraja radiata Annotation Release 100.20210331
mrpl49
XP_032872073.1
XM_033016182.1
scyl1
XP_032872087.1
XM_033016196.1
XM_033016186.1
XP_032872077.1
cdca5
XM_033016184.1
XP_032872075.1
LOC116969322
XR_004410727.1
Genes, Ensembl Rapid release 2020-06
ENSARAG00005017548 [+14]
ENSARAG00005017863
ENSARAT00005028527.1
ENSARAP00005027209.1
ENSARAG00005017886
ENSARAP00005027286.1
ENSARAT00005028608.1
ENSARAG00005017848
ENSARAP00005027186.1
ENSARAT00005028504.1
RNA-seq exon coverage, aggregate (filtered), NCBI Amblyraja radiata Annotation Release 100 - log 2 scaled

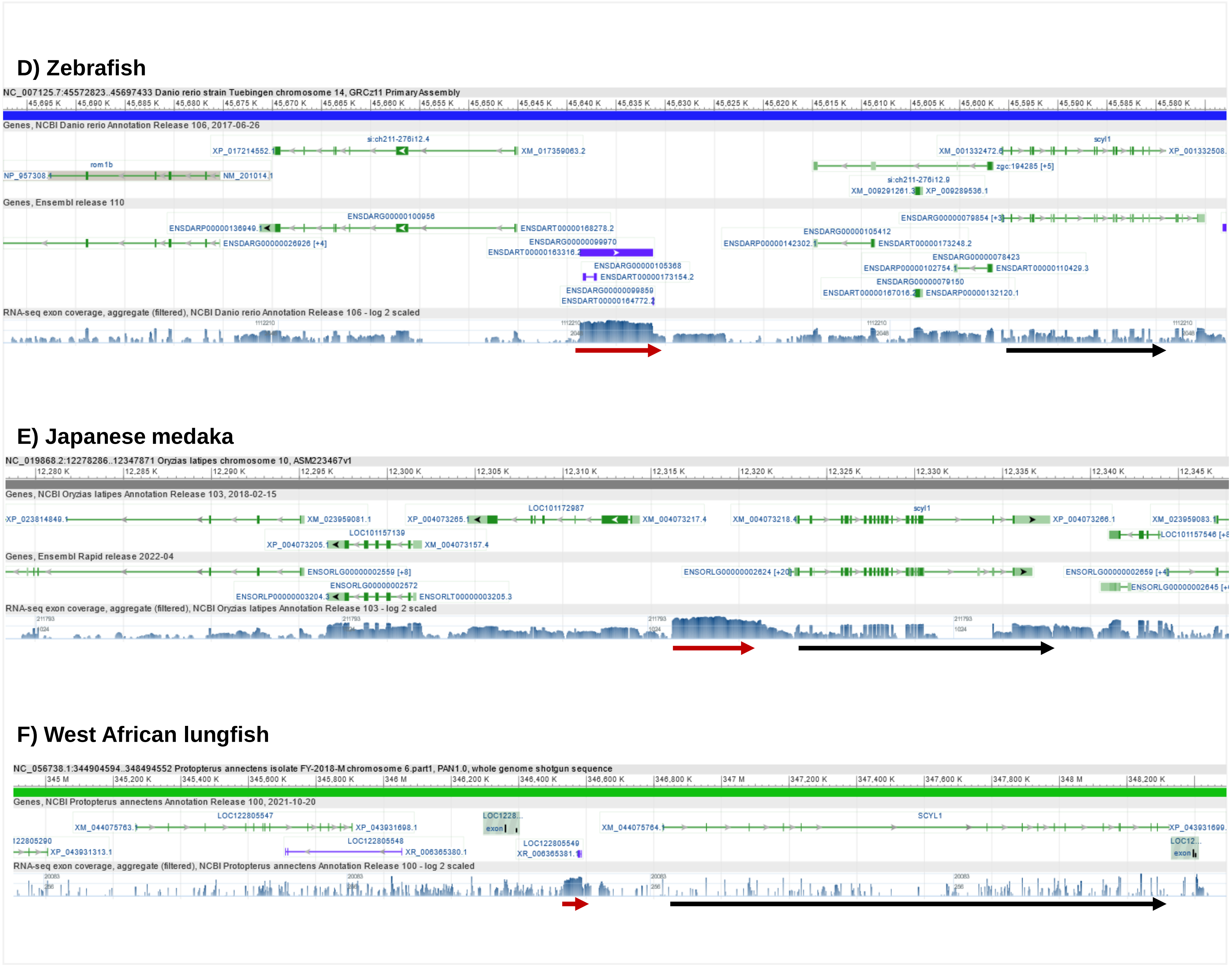
D) Zebrafish
NC_007125.7:45572823..45697433 Danio rerio strain Tuebingen chromosome 14, GRCz11 Primary Assembly
Genes, NCBI Danio rerio Annotation Release 106, 2017-06-26
Genes, Ensembl release 110
RNA-seq exon coverage, aggregate (filtered), NCBI Danio rerio Annotation Release 106 - log 2 scaled
E) Japanese medaka
NC_019868.2:12278286..12347871 Oryzias latipes chromosome 10, ASM223467v1
Genes, NCBI Oryzias latipes Annotation Release 103, 2018-02-15
Genes, Ensembl Rapid release 2022-04
RNA-seq exon coverage, aggregate (filtered), NCBI Oryzias latipes Annotation Release 103 - log 2 scaled
F) West African lungfish
NC_056738.1:344904594..348494552 Protopterus annectens isolate FY-2018-M chromosome 6.part1, PAN1.0, whole genome shotgun sequence
Genes, NCBI Protopterus annectens Annotation Release 100, 2021-10-20
RNA-seq exon coverage, aggregate (filtered), NCBI Protopterus annectens Annotation Release 100 - log 2 scaled

G) Coelacanth

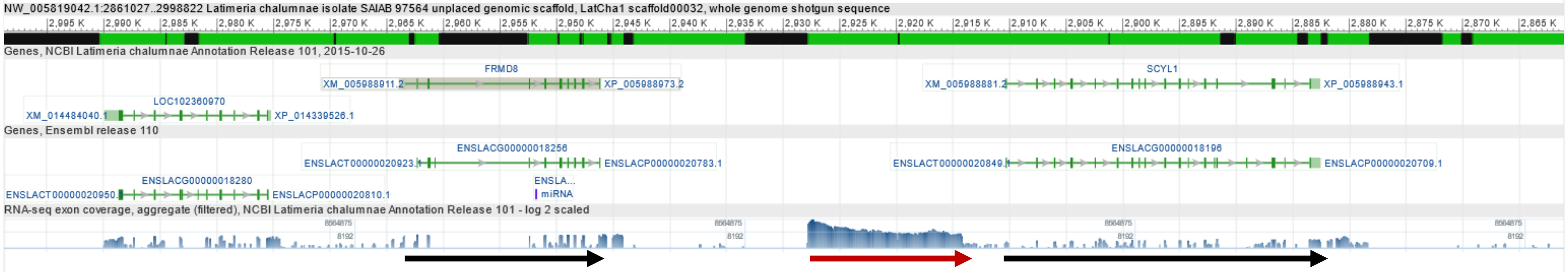

H) Tropical clawed frog

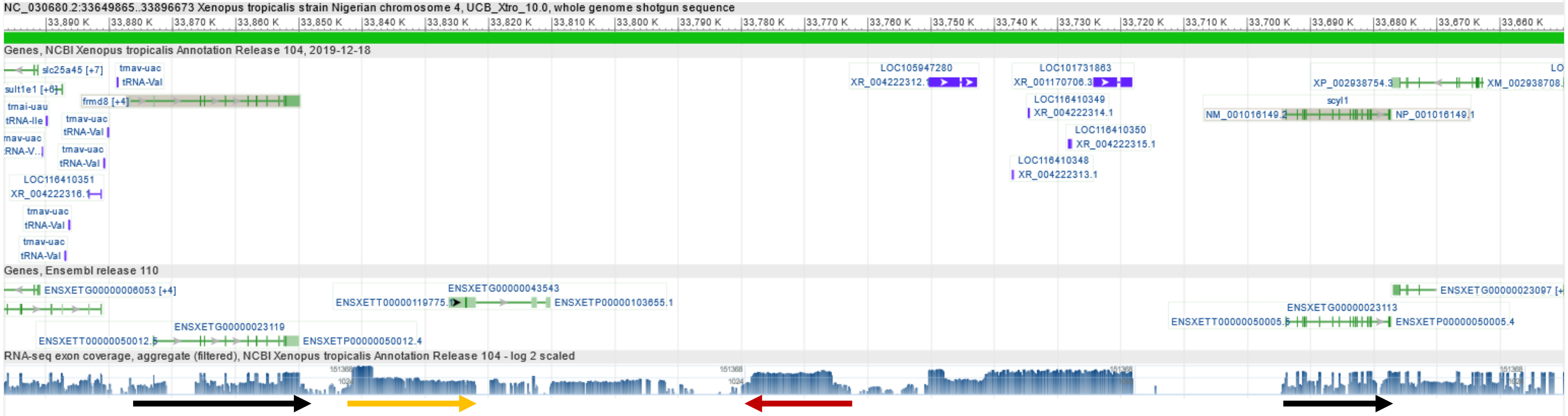

I) Common Toad

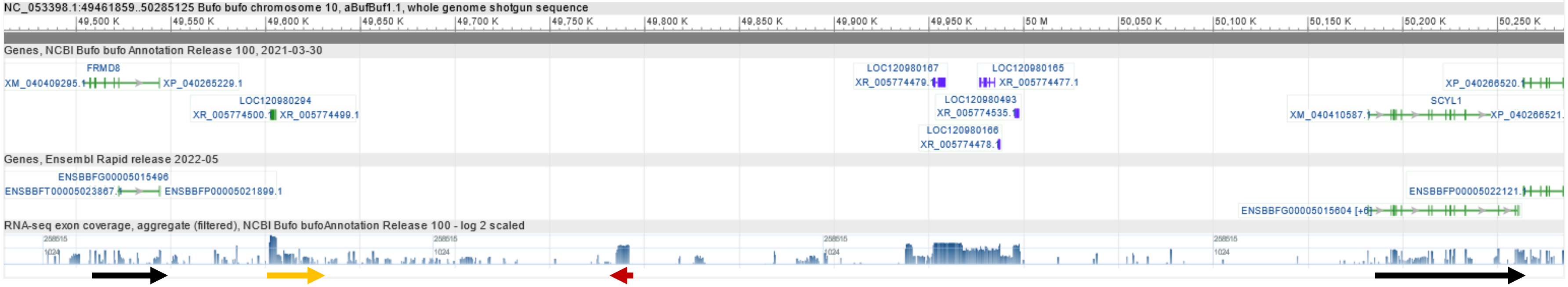

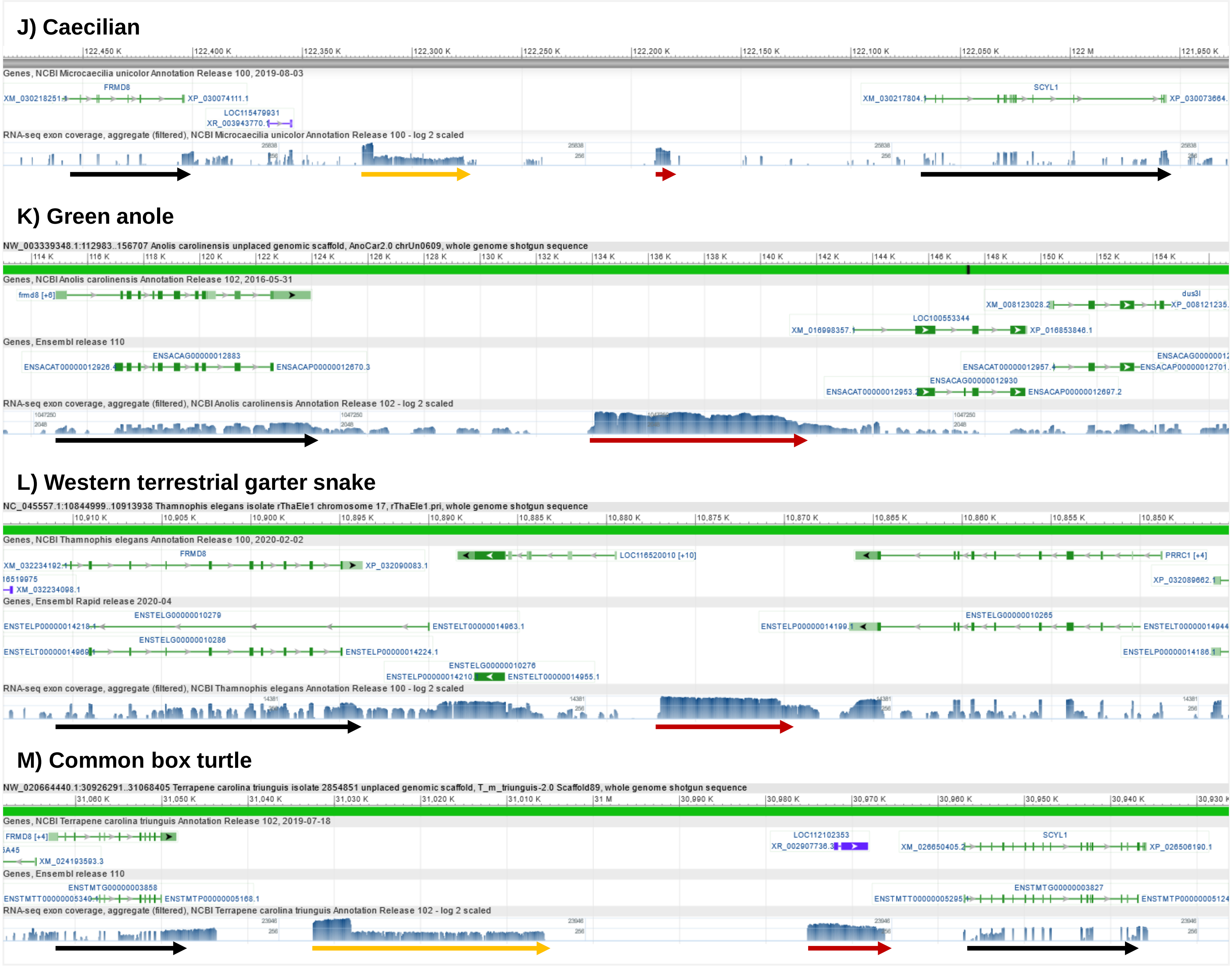

J) Caecilian
Genes, NCBI Microcaecilia unicolor Annotation Release 100, 2019-08-03
FRMD8
XM_030218251.1
XP_030074111.1
LOC115479931
XR_003943770.1
SCYL1
XM_030217804.1
XP_030073664.1
RNA-seq exon coverage, aggregate (filtered), NCBI Microcaecilia unicolor Annotation Release 100 - log 2 scaled
K) Green anole
NW_003339348.1:112983..156707 Anolis carolinensis unplaced genomic scaffold, AnoCar2.0 chrUn0609, whole genome shotgun sequence
Genes, NCBI Anolis carolinensis Annotation Release 102, 2016-05-31
frmd8 [+6]
dus3l
XM_008123028.2
XP_008121235.1
LOC100553344
XM_016998357.1
XP_016853846.1
Genes, Ensembl release 110
ENSACAG00000012883
ENSACAT00000012926.4
ENSACAP00000012670.3
ENSACAT00000012957.4
ENSACAP00000012701.3
ENSACAG00000012930
ENSACAT00000012953.2
ENSACAP00000012697.2
RNA-seq exon coverage, aggregate (filtered), NCBI Anolis carolinensis Annotation Release 102 - log 2 scaled
L) Western terrestrial garter snake
NC_045557.1:10844999..10913938 Thamnophis elegans isolate rThaEle1 chromosome 17, rThaEle1.pri, whole genome shotgun sequence
Genes, NCBI Thamnophis elegans Annotation Release 100, 2020-02-02
FRMD8
XM_032234192.1
XP_032090083.1
LOC116520010 [+10]
PRRC1 [+4]
XM_032234098.1
XP_032089662.1
Genes, Ensembl Rapid release 2020-04
ENSTELG00000010279
ENSTELP00000014218.1
ENSTELT00000014963.1
ENSTELG00000010285
ENSTELP00000014199.1
ENSTELT00000014944.1
ENSTELG00000010286
ENSTELT00000014969.1
ENSTELP00000014224.1
ENSTELP00000014186.1
ENSTELG00000010276
ENSTELP00000014210.1
ENSTELT00000014955.1
RNA-seq exon coverage, aggregate (filtered), NCBI Thamnophis elegans Annotation Release 100 - log 2 scaled
M) Common box turtle
NW_020664440.1:30926291..31068405 Terrapene carolina triunguis isolate 2854851 unplaced genomic scaffold, T_m_triunguis-2.0 Scaffold89, whole genome shotgun sequence
Genes, NCBI Terrapene carolina triunguis Annotation Release 102, 2019-07-18
FRMD8 [+4]
XM_024193593.3
LOC112102353
XR_002907736.3
SCYL1
XM_026650405.2
XP_026506190.1
Genes, Ensembl release 110
ENSTMTG00000003858
ENSTMTT00000005340.1
ENSTMTP00000005168.1
ENSTMTG00000003827
ENSTMTT00000005295.1
RNA-seq exon coverage, aggregate (filtered), NCBI Terrapene carolina triunguis Annotation Release 102 - log 2 scaled

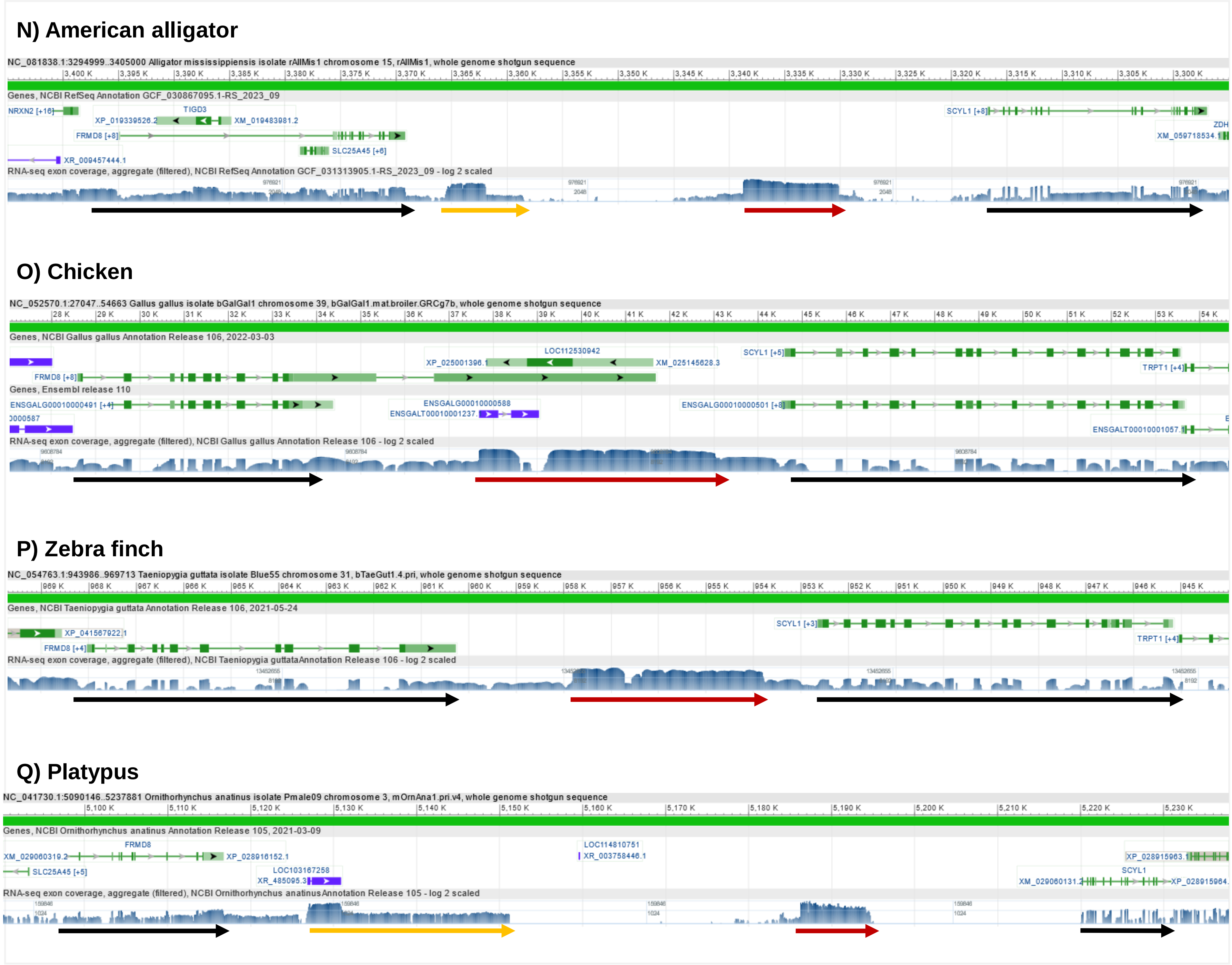

N) American alligator
NC_081838.1:3294999..3405000 Alligator mississippiensis isolate rAllMis1 chromosome 15, rAllMis1, whole genome shotgun sequence
Genes, NCBI RefSeq Annotation GCF_030867095.1-RS_2023_09
NRXN2 [+16]
TIGD3
XP_019339526.2
XM_019483981.2
FRMD8 [+8]
SLC25A45 [+6]
XR_009457444.1
SCYL1 [+8]
ZDH
XM_059718534.1
RNA-seq exon coverage, aggregate (filtered), NCBI RefSeq Annotation GCF_031313905.1-RS_2023_09 - log 2 scaled
O) Chicken
NC_052570.1:27047..54663 Gallus gallus isolate bGalGal1 chromosome 39, bGalGal1.mat.broiler.GRCg7b, whole genome shotgun sequence
Genes, NCBI Gallus gallus Annotation Release 106, 2022-03-03
LOC112530942
XP_025001396.1
XM_025145628.3
SCYL1 [+5]
FRMD8 [+8]
TRPT1 [+4]
Genes, Ensembl release 110
ENSGALG00010000491 [+4]
ENSGALG00010000588
ENSGALT00010001237.
ENSGALG00010000501 [+8]
ENSGALT00010001057.1
RNA-seq exon coverage, aggregate (filtered), NCBI Gallus gallus Annotation Release 106 - log 2 scaled
P) Zebra finch
NC_054763.1:943986..969713 Taeniopygia guttata isolate Blue55 chromosome 31, bTaeGut1.4.pri, whole genome shotgun sequence
Genes, NCBI Taeniopygia guttata Annotation Release 106, 2021-05-24
XP_041567922.1
FRMD8 [+4]
SCYL1 [+3]
TRPT1 [+4]
RNA-seq exon coverage, aggregate (filtered), NCBI Taeniopygia guttata Annotation Release 106 - log 2 scaled
Q) Platypus
NC_041730.1:5090146..5237881 Ornithorhynchus anatinus isolate Pmale09 chromosome 3, mOrnAna1.pri.v4, whole genome shotgun sequence
Genes, NCBI Ornithorhynchus anatinus Annotation Release 105, 2021-03-09
FRMD8
XM_029060319.2
XP_028916152.1
SLC25A45 [+5]
LOC103167258
XR_485095.3
LOC114810751
XR_003758446.1
XP_028915963.1
SCYL1
XM_029060131.2
XP_028915964.
RNA-seq exon coverage, aggregate (filtered), NCBI Ornithorhynchus anatinus Annotation Release 105 - log 2 scaled

R) Koala

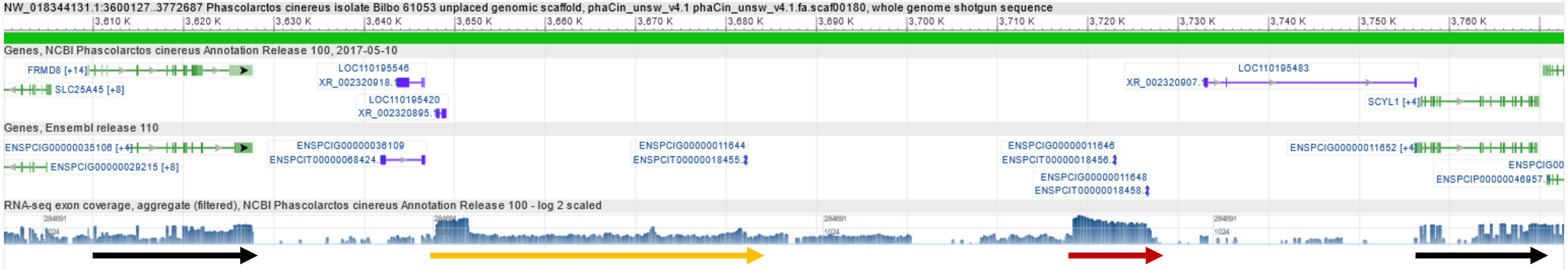

S) Human

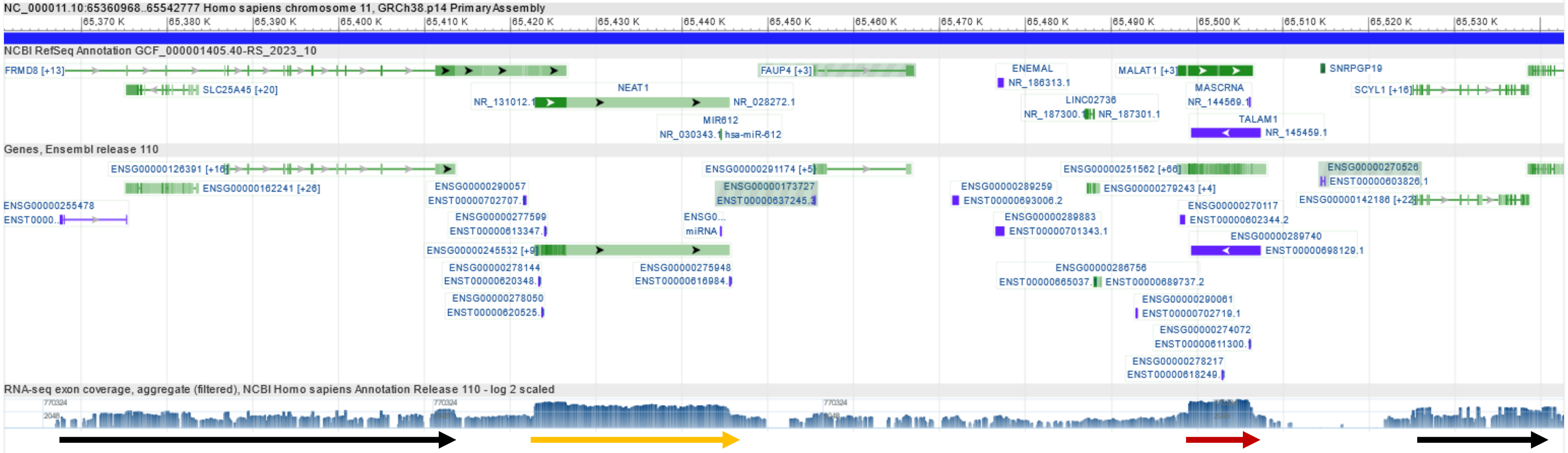

Supplement: Supplementary file 4 — Supplementary Fig. 1. RNA-seq coverage data of MALAT1 and NEAT1 in species representative of major vertebrate taxa. The genomic neighborhood surrounding MALAT1 and NEAT1 in species representative of major vertebrate taxa was exported from NCBI Genome Data Viewer. Tracks included current gene definitions in NCBI RefSeq and Ensembl genome assemblies, as well as RNA-seq alignment coverage. Arrows beneath the RNA-seq coverage track indicate the locations and strand of MALAT1 (red arrow), NEAT1 (orange arrow), and FRMD8 and/or SCYL1 (black arrows). For MALAT1 and NEAT1, the location of the arrowhead indicates the approximate location of the triple helix. The distinction between MALAT1 and NEAT1 can be observed in their RNA-seq coverage patterns for the species that have both. RNA-seq experiments that contributed to the coverage data for each species can be found in Supplementary Data 3 Supplementary file4 (PDF 1648 KB) [file 239_2023_10151_MOESM4_ESM.pdf]
